# Intravesical recurrence factors and outcome after radical nephroureterectomy for upper tract urothelial carcinoma: Multivariate analysis with propensity score matching

**DOI:** 10.3389/fonc.2022.984014

**Published:** 2022-08-18

**Authors:** Hang Zhao, Binbin Jiao, Kunpeng Liu, Zhenkai Luo, Zhenshan Ding, Shicong Lai, Jian Ren, Guan Zhang

**Affiliations:** ^1^ Department of Urology, China-Japan Friendship Hospital, Beijing, China; ^2^ China-Japan Friendship School Clinical Medicine, Peking University, Beijing, China; ^3^ Department of Urology, Beijing Chao-Yang Hospital, Capital Medical University, Beijing, China; ^4^ Beijing Laboratory of Biomedical Materials, State Key Laboratory of Organic-Inorganic Composite Materials, College of Life Science and Technology, Beijing University of Chemical Technology, Beijing, China; ^5^ Graduate School of Peking Union Medical College and Chinese Academy of Medical Sciences, Beijing, China; ^6^ Department of Colorectal Surgery, National Cancer Center/National Clinical Research Center for Cancer/Cancer Hospital, Chinese Academy of Medical Sciences and Peking Union Medical College, Beijing, China; ^7^ Department of Urology, Peking University People’s Hospital, Beijing, China

**Keywords:** intravesical recurrence, upper tract urothelial carcinoma, risk factor, oncological outcome, propensity score matching

## Abstract

**Objective:**

The risk factors for intravesical recurrence (IVR) after radical nephroureterectomy (RNU) in patients with upper tract urothelial carcinoma (UTUC) remain inconsistent and unclear. Thus, the risk factors of IVR after RNU and the prognostic significance of the risk indicators were explored herein.

**Methods:**

We retrospectively analyzed UTUC patients upon RNU in our center from January 2009 to December 2019. After propensity score matching, 139 patients were included in this study. Univariate and multivariate Cox proportional hazard regressions were used to estimate the hazard ratio and 95% confidence intervals. Overall survival (OS), cancer-specific survival (CSS) and recurrence-free survival (RFS) were measured using the Kaplan–Meier curve with a log-rank test. A *P*-value < 0.05 was considered statistically significant.

**Results:**

We included 139 patients with a median follow-up of 42 months, of which 48 patients had an intravesical recurrence. Multivariate Cox regression analysis showed cytological abnormalities in urine (HR=3.101, *P*=0.002), hydronephrosis (HR=1.852, *P*=0.042), adjuvant chemotherapy (HR=0.242, *P*<0.001), and previous history of bladder cancer (HR=5.51, *P*<0.001) were independent risk factors for IVR. As for clinical outcomes, OS and CSS suggested disadvantages in patients with IVR compared with patients without recurrence (*P*=0.042 for OS, *P*<0.0001 for CSS), OS of patients with abnormal urine cytology and OS and CSS of patients receiving adjuvant chemotherapy did not present clinical significance, and other risk factors all affected the clinical outcome.

**Conclusion:**

In this propensity-score matching study, cytological abnormality of urine, hydronephrosis, adjuvant chemotherapy and previous history of bladder cancer were shown to be independent risk factors for IVR. Moreover, risk factors also influence clinical outcomes, thereby rendering it necessary to adopt more active postoperative surveillance and treatment strategies for these patients, which may help improve treatment outcomes.

## Introduction

Upper urinary tract urothelial carcinoma (UTUC) consists of carcinoma of the renal pelvis and ureter, falling under the category of urothelial carcinoma together with bladder cancer. The disease mostly occurs at the age of 50 ~ 70 years old, accounting for 5% ~ 10% of all urothelial carcinoma (1). UTUC is more invasive and has a poor prognosis even after receiving the standard treatment of radical nephroureterectomy (RNU) and sleeve cystectomy in time, as 22% ~ 47% of patients still have an intravesical recurrence (IVR) during the follow-up period (2, 3). Therefore, how to identify the risk factors of IVR after RNU early and the factors that affect the prognosis of patients to individualize treatment has become a hot research topic in recent years.

Now, some studies have reported that tumor stage, grade, surgical method, lymphovascular invasion and other factors are independent risk factors for IVR after RNU (4, 5) besides the evidence revealing that the mortality of UTUC is relevant to the age, pathological grade and tumor stage (6, 7). In addition, with the gradual discovery of the role of inflammatory microenvironment in the occurrence and development of tumors, inflammatory indicators in the blood, including neutrophil-to-lymphocyte ratio (NLR), platelet-to-lymphocyte ratio (PLR) and lymphocyte- monocyte ratio (LMR), have also been proved to be related to the survival time and disease progression of tumor patients (8, 9), but there are still controversial about the impact of these factors.

Therefore, in order to analyze the risk factors of IVR after RNU and the prognostic significance of various clinical risk indicators, UTUC patients who underwent RNU in our center were retrospectively studied. Meanwhile, in a bid to minimize the impact of different groups of patients, we used propensity score matching (PSM) to achieve a balanced comparison (10).

## Methods

### Study population selection

This retrospective cohort study was approved by the institutional research ethics committee of our center (reference:2021-40-K24). Informed consent was obtained from all eligible participants in advance. This study was conducted and reported in accordance with STROCSS criteria (www.strocssguideline.com) (11).

The data involved in this retrospective study were collected prospectively from primary UTUC patients who underwent RNU in our center from January 2009 to December 2019

Inclusion criteria were: (1) patients with pathological diagnosis of UTUC; (2) patients with primary disease; (3) patients with unilateral onset; (4) patients subject to RNU combined with cystic sleeve resection.

Exclusion criteria were: (1) patients with bilateral UTUC; (2) patients subject to no RNU combined with cystectomy; (3) patients with metastatic uroepithelial carcinoma.

### Propensity score matching

**Figure 1:**
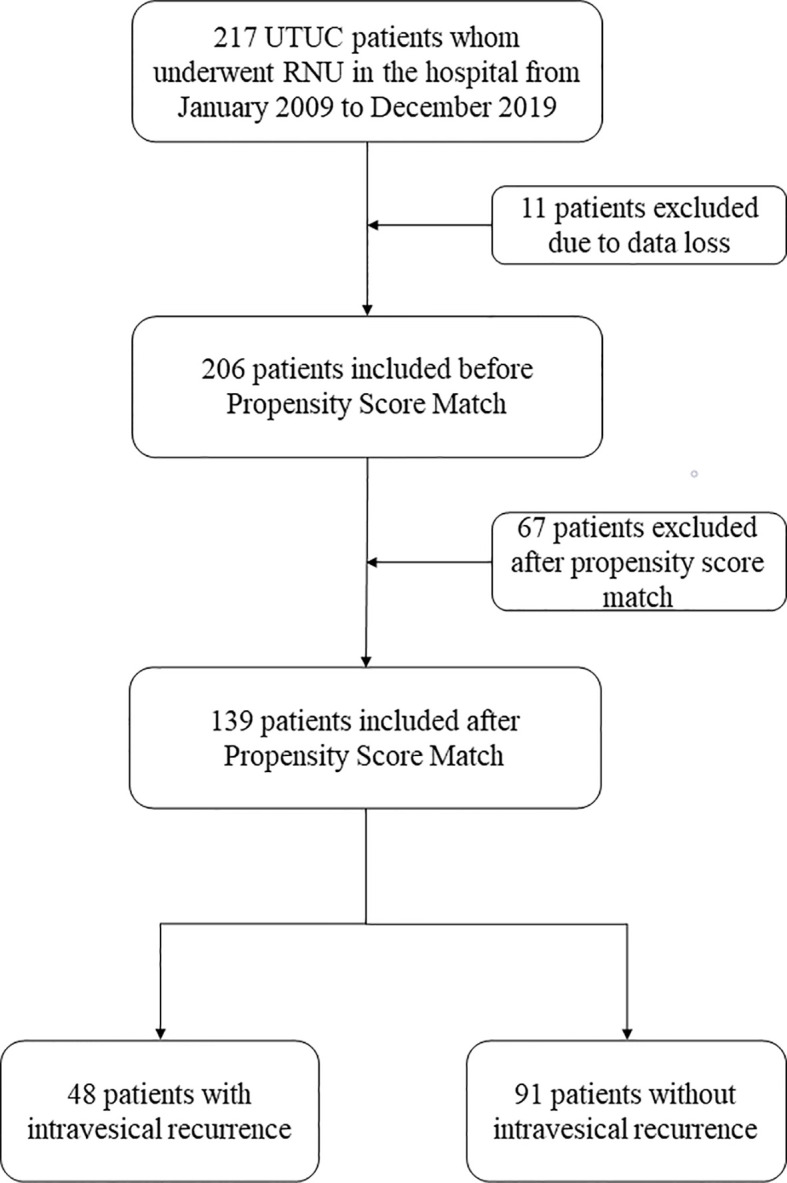
Flowchart of this study. UTUC, upper urinary tract urothelial carcinoma; RNU, radical nephroureterectomy.

A total of 217 patients were analyzed in this study. These patients underwent laparoscopic RNU with bladder cuff resection surgery. With 11 patients excluded due to data loss and 206 patients included in this analysis, we used the PSM method to adjust the confounders between the IVR and non-IVR groups. In our study, the propensity scores (PS) were based on variables such as age, sex, history of hypertension, diabetes mellitus, body mass index (BMI) and tumor side. A 1:2 greedy nearest neighbors matching method was applied, within PS calipers of ± 0.02, finally 48 patients with IVR and 91 patients with ono-IVR were included (**Figure 1**).

### Data collection and follow up

We collected the following detailed baseline information such as age, gender, tumor characteristics (including growth site, grade, stage, multifocality, architecture, peripheral lymphatic vascular invasion, etc.), blood-based inflammation biomarkers, including NLR, PLR, and LMR, serum creatinine, treatment plan, previous history of hypertension and diabetes, BMI, preoperative urine cytology, presence of hydronephrosis on the affected side, history of bladder cancer, early postoperative instillation, adjuvant chemotherapy, whether diagnostic ureteroscopy (URS) or retrograde intrarenal surgery was performed before RNU and long-term prognosis of all patients. All resected tumor specimens are generally staged according to the 2009 Union Internationale Contre le Cancer (UICC)/American Joint Committee on Cancer (AJCC) TNM classification (12). Tumor grade is determined conforming to the 2016 World Health Organization classification (13). Serum creatinine, NLR, PLR, and LMR was obtained from a routine blood examination performed 1 week before surgery. We defined hydronephrosis as the presence of hydrocephalus when the cortex and medulla of the affected side of the kidney were separated by more than 2cm on Computed Tomography (CT) before operation, renal dysfunction when the serum creatinine was more than 106 μmol/L, and abnormal urine cytology in the case of the presence of tumor cells or heterologous cells in preoperative urine routine examination. Intravesical instillation chemotherapy immediately or within 24 hours after RNU as early postoperative intravesical instillation chemotherapy, the chemotherapy regimen used included a single dose of mitomycin or epirubicin.

In addition to the preoperative detailed baseline information, we also collected the intraoperative and postoperative outcomes of radical nephroureterectomy. The outcome during the operation includes the operation time, the amount of bleeding during the operation and whether there is damage to the surrounding organs. The postoperative outcome includes the recovery time of gastrointestinal function, the length of hospital stay, the occurrence of complications such as infection, urinary fistula, and the retention time of the drainage tube.

Postoperative follow-up generally covers interval history and physical examination, routine blood analysis, urine cytology, chest radiography and CT urograms. Cystoscopic evaluation and urinary cytology were performed every 3 months for the first 2 years following RNU and every 6 months thereafter until 5 years. Thereafter, check-ups were scheduled annually. Additional imaging such as chest CT, bone scans or PET-CT examination was also employed as and when clinically required. In the present study, OS was defined as the time from the date of operation to death from any cause, CSS as the time from the date of RNU to the date of cancer-specific mortality, and RFS as the time from the date of RNU to the date of the first recurrence on imaging examination.

### Statistical analysis

Data were analyzed in SPSS 26.0 for Windows (IBM, Armonk, NY, USA). Qualitative variables are presented as numbers and percentages and were compared using the standard Chi-square test. Non-normally distributed quantitative data were exhibited as medians with interquartile ranges (IQR) which were then compared using the Mann-Whitney U test. All P values were two-tailed, with the threshold of statistical significance set at 0.05. Multivariate Cox regression analysis was exerted to correct for possible confounders, hence evaluating each assessment tool at predicting IVR. OS, CSS, and RFS were measured by virtue of the Kaplan–Meier method with a log-rank test with R software (version 4.1.2). A two-tailed *P*-value < 0.05 was considered statistically significant.

## Results

### Propensity score matching

In the study, the median follow-up time was 41 months (24,67.75), for the 206 eligible patients with a median age of 67 years, including 90 males and 116 females. Of the 206 patients, 51 (24.8%) registered IVR during the follow-up. After PSM, only 139 patients, including 61 males and 78 females, were included in the final study. Their median follow-up time was 42 months (25,64.5), and their median age was 69 years. Of the 139 patients, 48 (34.5%) suffered IVR (**Table 1**). Moreover, in the comparison between the IVR group and non-IVR group with the median follow-up time of 42 months, except for the significant differences (*P*<0.001) in preoperative urine cytology and adjuvant chemotherapy, the remaining clinical indicators, containing tumor location, stage, grade, size, architecture, past medical history, hydronephrosis and blood inflammatory indicators, showed no clinical differences (**Table 2**). And there was no significant difference between the IVR group and the non-IVR group in terms of intraoperative and postoperative outcomes (**Table S1**).

**Table 1:** Demographic and clinicopathological characteristics of patients with UTUC.

Variables	Propensity before total	Propensity after total
Number of patients	206	139
Follow-up period, mo median (range)	41 (24,67.75)	42 (25,64.5)
Sex (male/female)	90/116	61/78
Age, median (range)	67 (63.5,71.5)	69 (62,75)
Location, n (%)		
Renal pelvis	95 (46.1%)	62 (44.6%)
Ureter	104 (50.5%)	74 (53.2%)
Multifocality	7 (3.4%)	3 (2.2%)
Grade, n (%)		
Low grade	20 (9.7%)	13 (9.4%)
High grade	186 (90.3%)	126 (90.6%)
LVI, n (%)	7 (3.4%)	4 (2.9%)
Stage, n (%)		
pT1	103 (50.0%)	69 (49.6%)
pT2	51 (24.7%)	36 (25.9%)
pT3	44 (21.4%)	29 (20.9%)
pT4	8 (3.9%)	5 (3.6%)
Tumor diameter, n (%)		
<2cm	50 (24.3%)	33 (23.7%)
≥2cm	156 (75.7%)	106 (76.3%)
Tumor side (left/right)	111/95	65/74
History of hypertension, n (%)		
Present	104 (50.5%)	81 (58.3%)
Absent	102 (49.5%)	58 (41.7%)
History of diabetes, n (%)		
Present	41 (19.9%)	24 (17.3%)
Absent	165 (80.1%)	115 (82.7%)
BMI, median (range)	24.5 (22.1,27.0)	23.94 (21.85,26.43)
Urine cytology, n (%)		
Abnormal	119 (57.8%)	80 (57.6%)
Normal	87 (42.2%)	59 (42.4%)
Hydronephrosis, n (%)		
Present	75 (36.4%)	55 (39.6%)
Absent	131 (63.6%)	84 (60.4%)
Adjuvant chemotherapy, n (%)		
Present	104 (50.5%)	72 (51.8%)
Absent	102 (49.5%)	67 (48.2%)
Early postoperative instillation, n (%)		
Present	64 (31.1%)	29 (20.9%)
Absent	142 (68.9%)	110 (79.1%)
Renal dysfunction, n (%)		
Present	65 (31.6%)	44 (31.7%)
Absent	141 (68.4%)	95 (68.3%)
Tumor architecture, n (%)		
Papillary	48 (23.3%)	32 (23.0%)
Non-papillary	158 (76.7%)	107 (77.0%)
Previous history of bladder cancer, n (%)		
Present	11 (5.3%)	8 (5.8%)
Absent	195 (94.7%)	131 (94.2%)
URS, n (%)		
Present	122 (59.2%)	85 (61.2%)
Absent	84 (40.8%)	54 (38.8%)
IVR		
Present	51 (24.8%)	48 (34.5%)
Absent	155 (75.2%)	91 (65.5%)
NLR, median (range)	2.46 (1.88,3.32)	2.48 (1.91,3.68)
PLR, median (range)	134.26 (106.42,170.52)	136.84 (106.49,176.71)
LMR, median (range)	3.50 (2.50,4.67)	3.33 (2.42,4.59)

Values are given as median (IQR) or number (percentage); mo, month; LVI, lymphovascular invasion; BMI, body mass index; NLR, neutrophil-to-lymphocyte ratio; PLR, platelet-to-lymphocyte ratio; LMR, lymphocyte-monocyte ratio; URS, ureteroscopy.

**Table 2:** Demographic and clinicopathological characteristics of 139 patients after propensity score match.

Variables	With IVR	Without IVR	*P* value
Number of patients	48	91	
Follow-up period, mo median (range)	42 (25,65)	42 (25, 64.75)	0.852
Sex (male/female)	21/27	40/51	0.981
Age, median (range)	69 (62,75)	69 (62,75)	0.917
Location, n (%)			0.260
Renal pelvis	21 (43.8%)	44 (48.4%)	
Ureter	27 (56.2%)	47 (51.6%)	
Multifocality	0 (0%)	0 (0%)	
Grade, n (%)			0.765
Low grade	4 (8.3%)	9 (9.9%)	
High grade	44 (91.7%)	82 (90.1%)	
LVI, n (%)	1 (2.1%)	3 (3.3%)	0.685
Stage, n (%)			0.173
pT1	19 (39.6%)	50 (54.9%)	
pT2	17 (35.4%)	19 (20.9%)	
pT3	9 (18.8%)	20 (22.0%)	
pT4	3 (6.2%)	2 (2.2%)	
Tumor diameter, n (%)			0.781
<2cm	10 (20.8%)	23 (25.3%)	
≥2cm	38 (79.2%)	68 (74.7%)	
Tumor side (left/right)	22/26	43/48	0.874
History of hypertension, n (%)			0.992
Present	28 (58.3%)	53 (58.2%)	
Absent	20 (41.7%)	38 (41.8%)	
History of diabetes, n (%)			0.892
Present	8 (16.7%)	16 (17.6%)	
Absent	40 (83.3%)	75 (82.4%)	
BMI, median (range)	23.7 (21.8,26.4)	23.9 (21.9,26.4)	0.770
Urine cytology, n (%)			<0.001
Abnormal	38 (79.2%)	42 (46.2%)	
Normal	10 (20.8%)	49 (53.8%)	
Hydronephrosis, n (%)			0.069
Present	24 (50%)	31 (34.1%)	
Absent	24 (50%)	60 (65.9%)	
Early postoperative instillation, n (%)			0.079
Present	6 (31.1%)	23 (20.9%)	
Absent	42 (68.9%)	68 (79.1%)	
Adjuvant chemotherapy, n (%)			<0.001
Present	12 (25%)	60 (65.9%)	
Absent	36 (75%)	31 (34.1%)	
Renal dysfunction, n (%)			0.109
Present	11 (22.9%)	33 (36.3%)	
Absent	37 (77.1%)	58 (63.7%)	
Tumor architecture, n (%)			0.657
Papillary	10 (20.8%)	22 (24.2%)	
Non-papillary	38 (79.2%)	69 (75.8%)	
Previous history of bladder cancer, n (%)			0.088
Present	5 (10.4%)	3 (3.3%)	
Absent	43 (89.6%)	88 (96.7%)	
URS, n (%)			0.334
Present	32 (66.7%)	53 (58.2%)	
Absent	16 (33.3%)	38 (41.8%)	
NLR, median (range)	2.48 (1.91, 3.74)	2.48 (1.91,3.71)	0.323
PLR, median (range)	137.58 (107.47, 177.17)	137.21 (106.74,176.94)	0.607
LMR, median (range)	3.33 (2.41, 4.42)	3.33 (2.42,4.54)	0.302

Values are given as median (IQR) or number (percentage); mo, month; LVI, lymphovascular invasion; BMI, body mass index; NLR, neutrophil-to-lymphocyte ratio; PLR, platelet-to-lymphocyte ratio; LMR, lymphocyte-monocyte ratio; URS, ureteroscopy; Qualitative variables were compared by chi-square test and non-normally distributed quantitative variables were compared by Mann-Whitney U test.

### Cox regression analyses

In an attempt to explore independent risk factors of IVR after RNU, we performed univariate and multivariate Cox regression analyses on matched patients. Specifically speaking, the univariate Cox regression analysis showed that preoperative cytological abnormalities in urine (*P*<0.001), hydronephrosis (*P*=0.034), adjuvant chemotherapy (*P*<0.001) and previous history of bladder cancer (*P*=0.032) were risk factors for RNU; and the multivariate one evinced that preoperative cytological abnormalities in urine (HR=3.101,95%CI,1.503-6.398, *P*=0.002), hydronephrosis (HR=1.852,95%CI,1.022-3.356, *P*=0.042), adjuvant chemotherapy (HR=0.242,95%CI,0.123-0.437, *P*<0.001), and previous history of bladder cancer (HR=5.51,95%CI,2.050-14.811, *P*<0.001) were independent risk factors for IVR. However, elements like gender, tumor grade, stage, size, architecture, etc. did not show any clinical significance (**Table 3**).

**Table 3:** Univariate and multivariate Cox regression analyses predicting intravesical recurrence in 139 patients treated with radical nephroureterectomy for primary upper urinary tract urothelial carcinoma.

Variable	Univariate analysis			Multivariate analysis		
	HR	95% CI	*P*	HR	95% CI	*P*
Gender (male vs. female)	0.933	0.526-1.653	0.811			
Age (continuous)	1.004	0.973-1.037	0.799			
History of hypertension	1.021	0.574-1.817	0.944			
History of diabetes	1.112	0.519-2.385	0.784			
BMI (continuous)	0.982	0.897-1.075	0.69			
Urine cytology	3.725	1.840-7.542	<0.001	3.101	1.503-6.398	0.002
Hydronephrosis	1.85	1.047-3.268	0.034	1.852	1.022-3.356	0.042
renal dysfunction	0.584	0.298-1.146	0.118			
Tumor side (left vs. right)	1.055	0.597-1.863	0.854			
Tumor location (pelvis vs. ureteral)	1.325	0.870-2.018	0.527			
Tumor grade (low vs. high)	1.249	0.448-3.483	0.671			
Tumor diameter (<2 vs.≥2cm)	1.036	0.866-1.240	0.699			
Early postoperative instillation	0.480	0.203-1.231	0.067			
Adjuvant chemotherapy	0.252	0.131-0.485	<0.001	0.242	0.123-0.473	<0.001
Lymphovascular invasion	1.509	0.205-11.110	0.686			
Tumor stage						
pT1	reference	reference				
pT2	1.39	0.337-5.733	0.648			
pT3	1.899	0.439-8.212	0.391			
pT4	2.423	0.563-10.423	0.235			
Tumor architecture	0.867	0.431-1.741	0.688			
Previous history of bladder cancer	2.787	1.093-7.108	0.032	5.51	2.050-14.811	<0.001
URS	1.310	0.717-2.392	0.380			
NLR	1.085	0.990-1.188	0.08			
PLR	1.003	0.999-1.007	0.123			
LMR	0.906	0.749-1.095	0.308			

BMI, body mass index; NLR, neutrophil-to-lymphocyte ratio; PLR, platelet-to-lymphocyte ratio; LMR, lymphocyte-monocyte ratio; URS, ureteroscopy.

### Oncological outcomes

**Figure 2:**
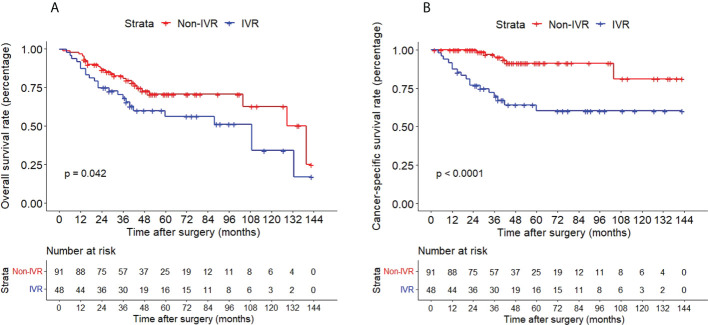
Kaplan–Meier curves for OS **(A)**, CSS **(B)** of patients with UTUC according to IVR. OS, overall survival; CSS, cancer-specific survival; UTUC, upper tract urothelial carcinoma; IVR, intravesical recurrence.

**Figure 3:**
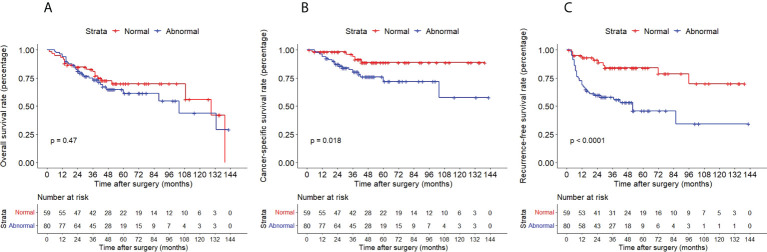
Kaplan–Meier curves for OS **(A)**, CSS **(B)** and RFS **(C)** of patients with UTUC according to urine cytology. OS, overall survival; CSS, cancer-specific survival; UTUC, upper tract urothelial carcinoma; IVR, intravesical recurrence.

**Figure 4:**
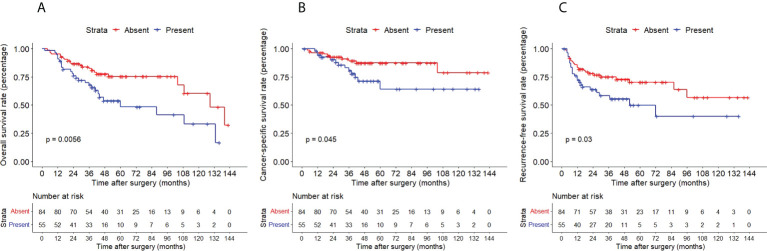
Kaplan–Meier curves for OS **(A)**, CSS **(B)** and RFS **(C)** of patients with UTUC according to hydronephrosis. OS, overall survival; CSS, cancer-specific survival; UTUC, upper tract urothelial carcinoma; IVR, intravesical recurrence.

**Figure 5:**
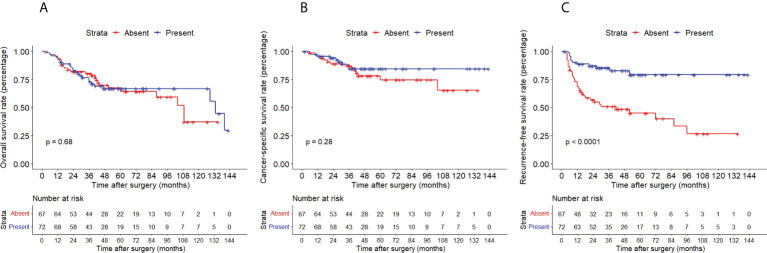
Kaplan–Meier curves for OS **(A)**, CSS **(B)** and RFS **(C)** of patients with UTUC according to adjuvant chemotherapy. OS, overall survival; CSS, cancer-specific survival; UTUC, upper tract urothelial carcinoma; IVR, intravesical recurrence.

**Figure 6:**
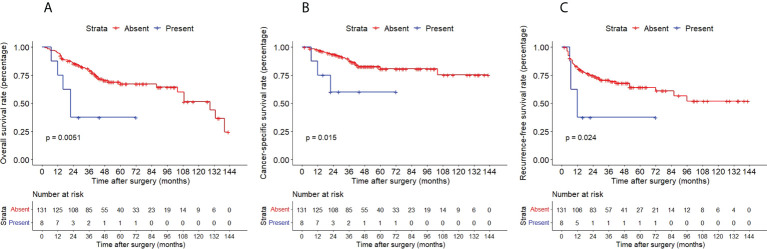
Kaplan–Meier curves for OS **(A)**, CSS **(B)** and RFS **(C)** of patients with UTUC according to previous history of bladder cancer. OS, overall survival; CSS, cancer-specific survival; UTUC, upper tract urothelial carcinoma; IVR, intravesical recurrence.

The Kaplan–Meier method with a log-rank test was exercised herein to evaluate the oncological outcomes between the IVR group and the non-IVR group, enabling us to explore whether the above factors had an impact on the oncological outcome based on the grouping of independent risk factors. The results showed that the two groups were significantly different in OS and CSS, with the OS and CSS of the IVR group significantly worse than those of the non-relapse group (*P*=0.042 for OS, *P*<0.0001 for CSS, **Figure 2**). Concerning urine cytology, CSS and RFS of patients with abnormal urine cytology were lower than those of normal patients, but OS displayed no significant difference (*P*=0.47 for OS, *P*=0.018 for CSS, *P*<0.0001 for RFS, **Figure 3**). OS, CSS and RFS of patients without hydronephrosis on preoperative imaging were significantly better than those with hydronephrosis (*P*=0.0056 for OS, *P*=0.045 for CSS, *P*=0.03 for RFS, **Figure 4**). With respect to patients receiving adjuvant chemotherapy, only RFS indicated clinical difference (*P*<0.0001), and neither OS nor CSS showed clinical significance (*P*=0.68 for OS, *P*=0.28 for CSS, **Figure 5**). Furthermore, there were significant disparities in OS, CSS and RFS between patients with and without a previous history of bladder cancer (*P*=0.0051 for OS, *P*=0.015 for CSS, *P*=0.024 for RFS, **Figure 6**).

## Discussion

As a relatively rare malignant tumor, the treatment of UTUC has registered tremendous progress in recent years. Nevertheless, further in-depth study about UTUC is still in urgent need given its high invasion and poor prognosis. Many studies have concluded that the risk factors of IVR after RNU include tumor stage, multifocality, preoperative ureteroscopic biopsy, male, previous history of bladder cancer, smoking, chronic kidney disease, tumor location, transurethral bladder cuff resection, positive resection margin, and surgical approach (4, 14–16), At the same time, there was also an article that has shown the independent role of biochemical parameters such as triglycerides to HDL cholesterol ratio and pseudocholinesterase levels in predicting the presence of bladder cancer (17). Whereas there are still different studies have put forward different views about it (18, 19), and there is still limited data on the development of prognosis of patients after RNU. In this context, we used the PSM method to minimize the interference of confounding factors, for the purpose of obtaining convincing risk factors of IVR in UTUC patients and the impact of these factors on the clinical outcome of UTUC patients after RNU.

The clinical data of 217 patients were under retrospective analysis in this study. After PSM, 139 patients were selected to construe the risk factors and clinical outcomes. Upon univariate and multivariate cox regression analysis, the results suggested that preoperative cytological abnormalities in urine (HR=3.101,95%CI,1.503-6.398, *P*=0.002), hydronephrosis (HR=1.852,95%CI,1.022-3.356, *P*=0.042), adjuvant chemotherapy (HR=0.242,95%CI,0.123-0.437, *P*<0.001) and previous history of bladder cancer (HR=5.51,95%CI,2.050-14.811, *P*<0.001) were proved risk factors for IVR. Apropos of clinical outcomes, OS and CSS showed disadvantages in patients with bladder recurrence compared with patients without recurrence (*P*=0.042 for OS, *P*<0.0001 for CSS). As to the influence of risk factors on clinical outcome, only OS of patients with abnormal urine cytology and OS, CSS of patients receiving adjuvant chemotherapy did not show clinical significance, and other risk factors all affected the clinical outcome.

Our results implied that abnormal urine cytology before operation was a risk factor for bladder recurrence after RNU, and patients would have more inferior CSS and RFS despite the normal OS. In the past decade, the intraluminal seeding and implantation hypothesis has been considered one of the mechanisms of postoperative recurrence (20), and the excretion of urine from the upper urinary tract is conducive to the implantation of tumor cells. There is a rational conclusion asserting that abnormal urine cytology before operation serves as a risk factor for recurrence in the bladder, which is consistent with the research of Kobayashi et al (21). Sakano et al. drew the same conclusion in their study of 536 patients (*P*=0.02 for urine cytology) that urine cytology is an independent predictor of disease-specific survival (22). Even though more studies focus on the early diagnostic value of urine cytology (23, 24), our study shows that abnormal urine cytology has a certain clinical value for postoperative recurrence and oncological outcome. Due to the small number of studies in this area, more studies are needed to further confirm the value of preoperative urine cytology.

In general, ureteral tumors can lead to progressive ureteral obstruction, resulting in hydronephrosis. Persistent obstruction leads to renal function damage; therefore, the degree of obstruction may be related to the tumor stage (25). By far, there are some research conclusions as follows. For example, Cho et al. reported that 86.0% of patients who have hydronephrosis grade 3 or 4 have invasive tumors (26); Ng et al. noted that hydronephrosis is independently associated with cancer metastasis and cancer-specific death (27); the retrospective analysis over 400 patients by Messer et al. validates that hydronephrosis is a valuable clinical predictor for advanced UTUC, also suggesting that simple radiographic assessment of presence or absence of hydronephrosis probably provides sufficient staging information alone but fails to correlate hydronephrosis with oncologic outcomes data owing to lack of oncologic follow-up data (25); Zhang et al. conducted univariate and multivariate analyses revealing that poorer CSS and OS are correlated with preoperative hydronephrosis (*P*=0.004 and *P*=0.009, respectively) (28). When it comes to the present study, our results clarified that preoperative hydronephrosis is an independent risk factor for postoperative recurrence, and OS, CSS and RFS showed clinical significance. Considering the mechanism between hydronephrosis and worse tumor outcome is still undiscovered, we can only infer that hydronephrosis may cause outward expansion and longitudinal thinning of the already narrow ureter or renal pelvis wall, which may facilitate the seeding of cancer cells to regional or distant organs, or increased outward centrifugal pressure causing reverse flow in lymphatics and vasculature, which can result in increased cancer seeding (29). Based on these findings herein, patients with preoperative hydronephrosis may need more active treatment and intervention.

According to the research of the British Association of Urological Surgeons Section of Oncology concluding that the incidence of bladder tumor recurrence in the first year after RNU is significantly reduced by administering a single postoperative dose of intravesical chemotherapy (30), Our results showed that early postoperative intravesical chemotherapy did not significantly reduce the probability of intravesical recurrence, which is different from the results. This may be due to the small number of patients and the different retention time of chemotherapy drugs in each patient’s bladder. Adjuvant systemic chemotherapy for UTUC may inhibit IVR of urothelial cancer, which is consistent with our results, but our results further showed that adjuvant chemotherapy has no clinical significance for patients’ OS and CSS. Hellenthal and colleagues also find no significant differences in 5-year OS or CSS — 38% and 45% respectively in 542 UTUC patients who underwent RNU. But the retrospective analysis of 245 patients by Lo et al. proposed that adjuvant chemotherapy is beneficial to OS and disease-free survival (DFS) (31), and Gregg et al. performed a meta-analysis indicating that perioperative chemotherapy is associated with improved OS (HR 0.75, 95%CI 0.57–0.99), DFS (HR 0.54, 95%CI 0.32-0.92), and CSS (HR 0.69, 95%CI 0.42–1.15), however, since all the included studies were retrospective and 2 literature included neoadjuvant chemotherapy, the effect of adjuvant chemotherapy may create a certain bias (32). Our retrospective analysis included patients over the past decade, during which the supportive treatment methods and systematic treatment views were renewed with the development of surgery, possibly serving as the potential sources of negative results. Moreover, we included 44 patients with preoperative abnormal renal function, which may limit the application of adjuvant chemotherapy and produce negative results.

The meta-analysis of Seisen et al. demonstrated that previous bladder cancer (HR 1.96, 95% CI 1.73–2.22; *P*<0.001) is a significant predictor of IVR (14), which is congruent with the conclusions of many studies (5,4,29), confirming that metachronous urothelial carcinomas, arising either from the upper or lower urinary tract, might be caused by distinct transformed cells that have acquired individual genetic alterations (33). Our results also support this view, and the clinical outcomes also show that the previous history of bladder cancer has an impact on the prognosis of patients, which coincides with the results of Ku et al. (34). Nonetheless, there are only 8 patients with previous bladder cancer in this study, which may cause some deviation in results. More large-scale studies are needed to verify the impact of the previous history of bladder cancer on oncological outcomes.

The advantage of this study is that the PSM method is used to eliminate the influence of confounding factors as much as possible, thus making the research results substantially credible. But there are several limitations to the study that should be acknowledged. First, this is a retrospective study with limited sample size, especially after PSM, only 139 patients were included; therefore, more prospective studies or randomized controlled trials in the future are needed to verify the conclusion drawn here. Second, the time span was relatively long, and the standards of blood tests, urine exfoliated cytology, and chemotherapy regimen may have changed during this study period, further affecting the reliability of the collected data. Third, regarding the data from a single center, the limited amount of cases restricted further analyses, which may require stratification of certain parameters.

## Conclusion

Our study suggested that the preoperative cytological abnormality of urine, hydronephrosis, adjuvant chemotherapy and previous history of bladder cancer were independent risk factors for IVR after RNU. Meanwhile, some of these risk factors will also pose a significant impact on patients’ OS, CSS and RFS, indicating that more active intervention, treatment and follow-up are required for these patients. It is also our sincere wish that the findings of this study will be confirmed by further prospective multicenter trials.

## Data availability statement

All the data were presented in the manuscript. No additional data are available. Requests to access the datasets should be directed to GZ, gzhang2016@sina.com.

## Ethics statement

The studies involving human participants were reviewed and approved by China-Japan Friendship Hospital. The patients/participants provided their written informed consent to participate in this study.

## Author contributions

HZ and BJ conceived of the presented idea. HZ and BJ developed the theory and performed the computations. KL, ZD, and ZL verified the underlying data. BJ, HZ, ZD, SL, KL, ZL, and JR collected the data, GZ supervises this study. All authors discussed the results and contributed to the final manuscript.

## Funding

This research did not receive any specific grant from funding agencies in the public, commercial, or not-for-profit sectors.

## Conflict of interest

The authors declare that the research was conducted in the absence of any commercial or financial relationships that could be construed as a potential conflict of interest.

The authors have fully disclosed their conflicts of interest in the manuscript and during the submission process. This manuscript has not been published and is not under consideration for publication elsewhere.

## Publisher’s note

All claims expressed in this article are solely those of the authors and do not necessarily represent those of their affiliated organizations, or those of the publisher, the editors and the reviewers. Any product that may be evaluated in this article, or claim that may be made by its manufacturer, is not guaranteed or endorsed by the publisher.
