# Supplementary material for: Intravesical recurrence factors and outcome after radical nephroureterectomy for upper tract urothelial carcinoma: Multivariate analysis with propensity score matching
Source: Front Oncol. 2022 Aug 18;12:984014. doi: 10.3389/fonc.2022.984014 (PMC9433701; doi:10.3389/fonc.2022.984014)
Supplement: Supplementary file 1 [file Table_1.docx]

**Table S1. Intraoperative and postoperative outcomes in 139 patients treated with radical nephroureterectomy for primary upper urinary tract urothelial carcinoma.**

| Variables | With IVR | Without IVR | *P* value |
| --- | --- | --- | --- |
| Number of patients | 48 | 91 |  |
| **Intraoperative outcome** |  |  |  |
| Operation time, min median (range) | 114 (104.5,135.25) | 118 (106,129.5) | 0.667 |
| The amount of bleeding, ml median (range) | 183 (105.75,248.25) | 222 (140,282) | 0.195 |
| Damage to surrounding viscera, n (%) | 1 (2.1%) | 3 (3.3%) | 0.685 |
| **Postoperative outcome** |  |  |  |
| Gastrointestinal function, days median (range) | 3 (2,5.25) | 4 (3,5) | 0.216 |
| Length of hospital stay, days median (range) | 9 (8,12) | 10 (7,11.5) | 0.250 |
| Complications, n (%) | 4 (8.3%) | 3 (3.3%) | 0.198 |
| Retention time of the drainage tube, days median (range) | 5 (4,6) | 5 (4,6) | 0.562 |

Notes: Values are given as median (IQR) or number (percentage); min, minutes; ml, milliliter; Qualitative variables were compared by chi-square test and non-normally distributed quantitative variables were compared by Mann-Whitney U test.
